# India's progressive step: Regulating tobacco on Over-the-Top streaming platforms

**DOI:** 10.18332/tpc/181371

**Published:** 2024-01-25

**Authors:** Nancy Satpathy, Epari Venkata Rao, Pratap Kumar Jena

**Affiliations:** 1 Siksha 'O' Anusandhan, Deemed to be University, Bhubaneswar, India; 2 KIIT School of Public Health, Kalinga Institute of Industrial Technology, Bhubaneswar, India

**Keywords:** OTT, Tobacco, India

**Dear Editor,**

India has taken a commendable step by becoming the first country to regulate the promotion of tobacco in the content displayed on Over-the-Top (OTT) streaming platforms such as Netflix, Amazon Prime, Voot, ZEE5, Disney Hotstar, etc.^1^. The move is an extension of the existing provisions of the Cigarettes and Other Tobacco Products Act (COTPA), which previously focused on traditional media^2^. This dynamic move aims to curtail exposure to tobacco imagery in movies, short videos, and web series, hosted by the new age and popular OTT platforms and, in turn, can protect impressionable youth from the harmful influence of such depictions.

The decision to regulate tobacco on OTT platforms was prompted by an extensive research effort involving data mining, interviews, and in-depth analysis of movies and web series, available on popular streaming platforms. Multiple researches revealed an alarming trend wherein tobacco use scenes were depicted without statutory warnings or disclaimers, creating a 'cool' and 'glamorous' image of tobacco^3,4^. Such depictions violated the existing COTPA Act and had a concerning influence on vulnerable individuals, especially youth^5^. The comprehensive investigation and reporting played a pivotal role in highlighting the need for regulation within the OTT space. This development ensures that tobacco use scenes are appropriately addressed and necessary warnings are prominently displayed, safeguarding the well-being of viewers, especially the younger audience.

The newly introduced Cigarettes and Other Tobacco Products (Prohibition of Advertisement and Regulation of Trade and Commerce, Production, Supply, and Distribution) Amendment Rules, 2023, notified by the Ministry of Health and Family Welfare of India on World Tobacco Day, 31 May 2023 [GSR 400(E)]^1^, outlines stringent requirements for streaming platforms. These rules mandate displaying anti-tobacco health spots at the beginning and middle of the program, showing a prominent health warning message at the bottom of the screen while displaying tobacco products, and incorporating audio-visual disclaimers about the harmful effects of tobacco use. Failure to comply with these rules will result in action by an inter-ministerial committee, and publishers will be allowed to explain any non-compliance and make appropriate modifications to their content.

The impact of these stringent laws on the industry will be witnessed in the future. However, the clear intent to protect the health and well-being of the Indian population is the heart of this regulation. This significant development is poised to change the content landscape positively, promoting responsible storytelling. OTT platforms can actively discourage tobacco use and foster a healthier society by incorporating warnings, disclaimers, and health spots. India's pioneering move to regulate tobacco in OTT content, sets an exemplary standard for the world, demonstrating the country’s commitment to public health and responsible media practices.

## Data Availability

Data sharing is not applicable to this article as no new data were created.
